# Socio-economic inequalities in unmet long-term care needs in Spain

**DOI:** 10.1007/s10433-026-00910-3

**Published:** 2026-02-04

**Authors:** Raquel Andres, Alexandrina Stoyanova

**Affiliations:** 1 Department of Economics, Faculty of Economics and Business, Universitat de Barcelona, Av. Diagonal 696, 08034 Barcelona, Spain; 2 Barcelona Economic Analysis Team (BEAT), Universitat de Barcelona, Barcelona, Spain

**Keywords:** Long-term care, Care gap, Unmet needs, Inequalities, Concentration index

## Abstract

Europe’s rapidly ageing population places increasing pressure on long-term care (LTC) systems, with inequitable access standing out as a key challenge. While socio-economic disparities in LTC utilisation are well documented, inequalities in unmet needs—the gap between required and received care—are less studied. Using the 2019 European Health Interview Survey for Spain, we categorise unmet needs as fully unmet, partially unmet, or having no unmet needs and quantify the care gap in hours. We assess socio-economic inequalities and inequities in unmet needs, stratified by eligibility for publicly funded LTC and by limitation severity (number of limitations in activities of daily living). We observe that unmet LTC needs disproportionately affect women, individuals living alone, and the oldest old. In addition, we find inequality and inequity, with a disproportionate burden borne by poorer individuals and those with severe limitations. Our decomposition analysis reveals that health status, income, and living arrangement explain most of the observed inequality. Notably, among eligible individuals with more severe limitations, living arrangement emerges as the largest contributor, as having a spouse mitigates inequalities in unmet needs, while living alone exacerbates them. These results highlight the need to reduce inequalities that disproportionally affect the lowest socio-economic strata and to address disparities through more effective resource allocation and targeted policies. By using a continuous measure of unmet LTC need that allows for a more nuanced analysis of its socio-economic determinants, this study contributes to the broader debate on the fairness and efficiency of LTC provision in ageing societies.

**Supplementary Information:**

The online version contains supplementary material available at 10.1007/s10433-026-00910-3.

## Introduction

The rapid ageing of the European population presents a pressing challenge for health and social care systems. Long-term care (LTC) plays a crucial role in supporting older adults with limitations in daily activities, yet demographic and cultural shifts, such as the sharp rise in women’s labour-market participation and higher rates of family dissolution, have strained traditional informal care arrangements and increased pressure on welfare systems (Di Novi et al. 2015; Brenna and Di Novi 2015). Spain faces particularly striking demographic changes. By 2050, the population aged 65 and over is projected to rise from around 20% today to 30% (INE 2024). Almost one in two individuals aged 65 and over report difficulties with activities of daily living and around 30% present cognitive impairments (INE 2025). Given the rising demand for care, meeting these needs will be increasingly challenging. Unmet LTC needs are already prevalent, affecting around one-quarter of older adults and carrying adverse health and social consequences, including heightened risk of social exclusion (Rahman et al. 2022; Laferrère and Van den Bosch 2015). They are also associated with increased use of health care services (Sands et al. 2006; DePalma et al. 2013; Hass et al. 2017), declining health and rising mortality (He et al. 2015; Zhen et al. 2015). In this context, it is particularly relevant to assess the extent to which individuals with care needs receive the help they require.

‘Unmet need’ generally refers to a need for help that is not satisfied. However, its conceptualisation in LTC research varies widely (Spiers et al. 2022). These differences stem from divergent views on what constitutes care needs and, on the criteria used to assess whether needs are adequately addressed. Needs can be measured either through self-reported difficulties with daily activities in survey data (García-Gómez 2015; Ilinca et al. 2017; Hu et al. 2022) or through professionally assessed evaluations typically found in administrative records (Tenand et al. 2020; Vidiella-Martin et al. 2024). Care needs are most commonly assessed through limitations in activities of daily living (ADLs), such as eating, bathing, dressing, toileting, instrumental activities of daily living (IADLs), including cooking, shopping, managing finances, and taking medication, and mobility tasks. Regarding whether needs are satisfied or not, the literature distinguishes between *absolute* or *objective* unmet needs, where a person with care needs does not receive any help (Vlachantoni 2019; Albuquerque 2022; Calderón-Jaramillo and Zueras 2023) and *subjective* or *partially* unmet needs, where an individual is unsatisfied with the help received or needs more help (Kröger et al. 2019; Brimblecombe 2022).^1^ Our study considers a measure of unmet LTC needs that combines both the absolute and relative approaches (Zhang et al. 2025), classifying individuals into three categories: fully unmet needs (absolute), partially unmet needs (relative), and no unmet needs. This is then used to quantify unmet needs in hours by mapping functional and mental health limitations to hours of care required, while also accounting for each individual’s socio-economic characteristics.

Although quantifying unmet needs is relevant, our main research focus is on whether they are distributed unequally, that is, whether they disproportionately affect individuals in disadvantaged socio-economic positions in Spain. While inequalities and inequities in LTC utilisation are well documented (García-Gómez et al. 2015; Bakx et al. 2015; Carrieri et al. 2017; Lera et al. 2021; Floridi et al. 2021), inequalities in unmet needs remain comparatively understudied. Only three papers have explicitly analysed socio-economic disparities in unmet LTC needs using the concentration index (CI) approach (Wagstaff et al. 1989; Wagstaff and van Doorslaer 2000), making them the closest to our work. Hernández-Quevedo and Jiménez Rubio (2011) show inequity in unmet home-care needs disproportionately affecting disabled individuals at the bottom of the income distribution in Spain. García-Gómez et al. (2015) extend this work by distinguishing subjective and objective unmet needs and analysing different care types, showing that subjective unmet needs for home care and objective unmet needs are more concentrated among disadvantaged groups. Our study builds on this evidence by using more recent Spanish data and employing a continuous measure of unmet LTC needs that integrates both objective and subjective components. Moreover, we decompose the estimated inequalities into their determinants, which has not been previously done for Spain. Lastly, using UK data, Hu et al. (2022) report that absolute unmet needs disproportionally affect low-income individuals. However, their approach differs from ours in two key aspects: they focus on people with dementia and rely only on objective measures of unmet needs.

Socio-economic disparities in unmet LTC needs are also assessed using other empirical approaches and are thus also relevant to our work. Albuquerque (2022) reports that higher levels of income or wealth are associated with lower likelihood of experiencing absolute unmet needs in Italy and Greece, though not in Spain or Portugal. Brimblecombe (2022) similarly finds that unmet needs decrease with income. In contrast, two recent studies report no significant income gradient in unmet LTC needs (Szenkurök et al. 2024; Zhang et al. 2025).

Our findings show that the care gap disproportionately affects vulnerable groups—women, individuals living alone, and the oldest old—and is concentrated among the poor, particularly among those with greater care needs. The decomposition indicates that health status, income, and living arrangements are the main drivers of disparities, consistently contributing to inequality disfavouring the poor. Health status explains most inequality among those with care needs, while socio-economic factors become the key determinants among those with severe limitations.

This study contributes to the literature in three main ways. First, we quantify unmet LTC needs through a replicable approach applied to harmonised data from the 2019 European Health Interview Survey (EHIS) for Spain, which allows to capture not only whether needs are unmet but also the intensity of the care gap. Second, we apply the CI framework to assess socio-economic inequalities and inequities in unmet LTC needs, providing an updated picture of the current magnitude of the care gap after successive policy reforms in the Spanish LTC system. Third, we present the first decomposition of the CI for unmet LTC needs in Spain, identifying the specific mechanisms driving inequality in access to LTC.

The remainder of the paper is organised as follows. Sect. "Institutional framework" provides an overview of the Spanish LTC system. Sect. "Methodology" outlines the methodology. Sect. "Data and descriptive statistics" presents the data and descriptive statistics. Section 5 reports the results, Sect. "Discussion" and discusses their implications for LTC policy. Section "Conclusions" concludes.

## Institutional framework

The Spanish LTC system is grounded on Act 39/2006, which created the System for Autonomy and Care for Dependency (SAAD). This legal framework marked a significant policy shift, recognizing LTC as a social right and aiming for universal coverage for all individuals with dependency, regardless of age, gender, or other characteristics.^2^ Governance and regulation are decentralised, with substantial authority granted to Spain’s autonomous regions. While this allows adaptation to regional needs, it also requires intergovernmental coordination to ensure equity and minimum standards across territories (Costa-Font et al. 2025). Despite the normative and institutional advances introduced by SAAD, Spain’s public LTC spending remains below that of many Northern and Central European welfare states (OECD 2024), constraining its capacity to meet rising care demands. Consequently, the system continues to face challenges of financial sustainability, territorial equity, and the adequacy of service provision. These structural constraints provide the policy background against which unmet LTC needs, and their unequal distribution, must be understood.

Eligibility for publicly funded LTC benefits is determined through a standardised national assessment, classifying individuals into three dependency levels based on difficulty in performing essential daily activities. The evaluation covers limitations in ADLs and IALDs (hereafter jointly referred to as (I)ADLs), as well as mental health limitations. The assigned level defines the type, intensity, and mix of support an individual is entitled to under SAAD, including cash transfers and in-kind services. However, unlike other European countries, the Spanish model does not allow care needs to be quantified in hours, an aspect central to our approach.

Only three LTC systems in Europe (Austria, Lithuania and Luxemburg) link limitations directly to hours of care needs (Llena-Nozal et al. 2025). Among these, only Austria uses a nationally standardised needs assessments based on (I)ADLs and mental health limitations,^3^ similar to the Spanish system. Moreover, both countries define eligibility thresholds based on assessed care needs (points in Spain, hours in Austria) and exhibit comparable acceptance rates for LTC benefits (around 80% of applications in both countries). These similarities make the Austrian model particularly suitable for our purpose, as it enables us to quantify care needs in hours rather than replicate any specific national entitlement rule.

Finally, our approach relies on the reasonable assumption that individuals with identical (I)ADL and mental health limitations have similar care needs, independently of whether they live in Austria, Spain or another EU country. A similar, albeit more simplified, mapping of limitation severity into hours of LTC needs has also been used in previous cross-country comparative analysis (Oliveira Hashiguchi and Llena-Nozal 2020).

## Methodology

We assess socio-economic inequalities in access to LTC employing the CI to measure inequality in the distribution of unmet LTC needs (Wagstaff et al. 1989; Wagstaff and van Doorslaer 2000). The CI is computed using the following expression:

1 $$CI=\frac{2}{\overline{y} }cov\left({y}_{i},{R}_{i}\right)$$

where $cov(.,.)$ represents the covariance between unmet LTC needs in hours (${y}_{i}$) and the relative ranking of individuals by economic status (${R}_{i}$), proxied by income. The term $\overline{y }$ represents the average level of unmet LTC needs. Negative values of the index indicate that unmet LTC needs are disproportionately concentrated among the worse-off, while positive values suggest a concentration among the better-off. A non-significant CI indicates no evidence of income-related inequality.

Given the bounded nature of the unmet needs variable, we employ the Erreygers-corrected CI (Erreygers 2009), previously used in studies examining inequalities in unmet needs and care utilisation (Ilinca et al. 2017; Lera et al. 2021).

2 $$CCI=\frac{4\overline{y}}{{b }_{n}-{a}_{n}}CI$$

where ${a}_{n}$ and ${b}_{n}$ represent the minimum and maximum values of the monthly hours of unmet LTC needs.

A key advantage of the CI is that it can be decomposed into need- and non-need determinants (Wagstaff et al. 2003). We first estimate the following linear model:

3 $${y}_{i}=\alpha \sum_{k}{\beta }_{k}{x}_{ki}+\sum_{p}{\delta }_{p}{z}_{pi}+{u}_{i}$$

which assumes that $${y}_{i}$$ is a linear and additively separate function of need ($${x}_{k})$$ and non-need ($${z}_{p})$$ factors, $${\beta }_{k}$$ and $${\delta }_{p}$$ are their respective coefficients, and $$u$$ is the error term. Similarly, as shown by Van de Poel et al. (2012), the CCI can be rewritten as:

4 $$CCI=\frac{4}{{b}_{n}-{a}_{n}}\left(\sum_{k}{\beta }_{k}{\overline{x} }_{k}{CI}_{x}+\sum_{p}{\delta }_{p}{\overline{z} }_{p}{CI}_{z}+{GCI}_{u}\right)$$

where ${\overline{x} }_{k}$ and ${\overline{z} }_{p}$ are the mean of ${x}_{k}$ and ${z}_{p}$, respectively, and ${CI}_{x}$ and ${CI}_{z}$ are their concentration indices with respect to income. ${GCI}_{u}$ is the generalized CI of the error term, a residual term that reflects the socio-economic inequality associated to unobserved factors.

In essence, Eq. (4) shows the CCI as a weighted sum of the income-related CIs of the determinants of unmet needs, where the weights reflect how sensitive these needs are to variations in each variable (i.e., semi-elasticity).

While inequality analysis examines how unmet LTC needs are distributed across socio-economic groups, from a policy perspective it may also be useful to explore whether, and to what extent, the uneven concentration of unmet needs is associated with factors beyond need, such as income, education, or living arrangements. This is the focus of inequity analysis. The inequity index (CCI_non-need_) is defined as the difference between the CCI and the contribution corresponding to the need variables:

5 $${CCI}_{non-need}=CCI-\frac{4}{{b}_{n}-{a}_{n}}\sum_{k}{\beta }_{k}{\overline{x} }_{k}{CI}_{x}$$

The CCI_non-need_ ranges from − 1 to 1, with 0 indicating no inequity. Negative values indicate inequity disfavouring the poor, i.e., unmet care needs are more concentrated among poorer individuals after adjusting for need. Conversely, positives values suggest inequity favouring the poor.

To ensure robustness, we compute bias-corrected bootstrapped confidence intervals for the CCI estimates using 1,000 replications.

## Data and descriptive statistics

### Data source

We use data from the third wave of the EHIS, conducted in 2019–2020 for Spain. The EHIS is a cross-sectional survey carried out across EU countries to collect harmonised information on health status, health care utilisation, health determinants, and sociodemographic characteristics of individuals aged 15 and over residing in private households. Institutionalised population is not included in the survey.

### Sample

The Spanish 2019 EHIS data set includes 22,072 individuals aged 15 and above. Among the 7,194 adults aged 65 and above, we focus on those reporting difficulties with at least one (I)ADL (45.9%), i.e., individuals with care needs. The final analytic sample therefore consists of 3,306 individuals.

### Measures

#### Hours of long-term care needed

To estimate unmet LTC needs in hours, we begin by quantifying the hours of LTC needs. LTC needs are measured using EHIS variables that assess individuals' ability to perform basic personal care tasks (ADLs) and household tasks (IADLs). For each activity, respondents rate their limitation using four response categories: “No difficulty”, “Some difficulty”, “A lot of difficulty” or “Cannot do at all”. Based on these questions, we define a binary indicator of care needs equal to one for individuals reporting at least some difficulty with any personal care activity or household task.

We then assign individuals with LTC needs a corresponding number of care hours using the method proposed by Warum et al. (2025), which maps survey-reported limitations to Austria’s LTC assessment system (Table 1). Following this mapping, each limitation is assigned a specific number of care hours needed (e.g., an individual reporting difficulty dressing is assigned 20 h of care per month). Total monthly care needs are obtained by summing the hours associated with each reported limitation. Additionally, individuals with walking difficulties or severe limitations due to mental health problems^4^ receive an extra 5 and 45 h per month, respectively.

**Table 1:** Mapping of the Austrian care need assessment system to EHIS variables *Source*: Authors' own elaboration based on Warum et al. (2025)

Limitations	EHIS variable	Monthly hours
*Personal care activities (ADLs)*		
Feeding yourself	pc1a	30
Getting in and out of a bed or chair	pc1b	30
Dressing and undressing	pc1c	20
Using toilets	pc1d	30
Bathing or showering	pc1e	35
*Household activities (IADLs)*		
Preparing meals	ha1a	30
Using the telephone or managing money	ha1b, ha1g	10
Shopping	h1ac	10
Managing medication	ha1d	3
Doing light housework	ha1e	10
occasional heavy housework	ha1f	10
*Additional items (functional limitations and mental health)*		
Difficulty walking half a km on level ground without the use of any aid or walking up or down 12 steps	pl6, pl7	5
Severely limited due to mental health problems	hs3, g24	45

Variable g24 is extracted from the Encuesta Europea de Salud (INE 2020), which corresponds exactly to the Spanish EHIS sample; however, these variables have been removed from the EHIS version

#### Unmet LTC needs

**Fig. 1:**
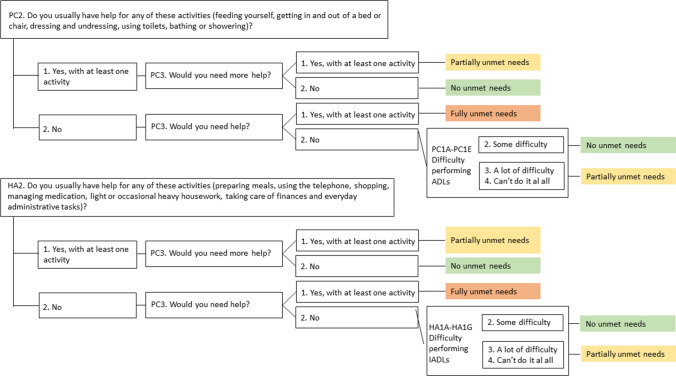
Classification of unmet LTC needs The classification is based on questions regarding the need for help due to limitations with personal care activities and household tasks in EHIS 2019. ADL: Activity of Daily Living. IADL: Instrumental Activity of Daily Living

We classify individuals as having no unmet needs (no gap) if they report difficulty with at least one (I)ADL, receive help with the corresponding activities, and state they do not need more help (see Fig. 1). Some respondents report difficulty, receive no help, yet state they do not need assistance; if all reported (I)ADL difficulties are mild (“some difficulty”), we also classify them as having no unmet needs, since we assume these limitations do not prevent independent performance of daily tasks. Individuals are classified as having fully unmet care needs (full gap) if they report difficulty with at least one (I)ADL, receive no support, and indicate needing help with at least one of these activities. Partially unmet needs (partial gap) correspond to situations in which respondents report difficulty, receive some help, but still require additional assistance, or cases in which they receive no help, report no need for help, but have severe limitations (“a lot of difficulty” or “can’t do it at all”). Finally, we translate each individual’s needs classification into estimated hours of unmet needs as follows: respondents classified as having fully unmet needs are assigned a care gap equal to the total estimated hours of care required; those in the no-gap category are assigned zero hours; and individuals with partially unmet needs are assigned a proportion of their estimated care hours based on a sliding-scale adjustment. This adjustment accounts for key contextual mitigators, including partnership status, co-residence, and receipt of formal care,^5^ aligned with evidence that living with a partner or family member reduces reported unmet needs (Pickard 2015; Ornstein et al. 2019; Bertogg and Strauss 2020; Zhang et al. 2025).

To assess robustness and limit potential bias introduced by the adjustment, we conduct a sensitivity analysis in which all partial-gap cases are assigned 50% of required hours as unmet need. This alternative specification yields comparable results.^6^

### Other variables

The other variables fall into two categories: need and non-need. Need variables include age, sex and health indicators. Sex is coded as binary; age is categorised into groups (65–69, 70–74, 75–79, 80–84, 85 +). Health indicators include self-reported health status (coded as 1 for less than good health, 0 otherwise), having a long-term health problem (yes/no), number of chronic conditions, number of ADLs and number of IADLs. The non-need variables are income, education, living arrangements, area of residence (urban/rural), and regional dummies. Our ranking variable is net monthly household income, adjusted for household size and composition using the OECD-modified equivalence scale. Because EHIS reports income only in quintiles, we use interval regression to impute continuous values.^7^ This imputation relies on the reported income quintile, the household income distribution from the European Union Statistics on Income and Living Conditions (EU-SILC) 2019 cross-sectional microdata (Eurostat 2019), and a set of covariates (age, sex, education, labour status, region, urban/rural residence, and family composition). This estimation was performed on the entire EHIS 2019 sample to maintain the original distribution of the income variable. Using a continuous ranking variable preserves individuals’ rank order and produce more accurate inequality estimates (Wagstaff et al. 1989). Education follows ISCED classification: low (level 2 or lower), medium (levels 3 and 4), and high (level 5 or higher). Partnership status reflects cohabitation with a legal or de facto partner. Since EHIS does not identify children outside the household, we proxy potential informal caregiving with a composite living arrangement variable derived from household size and partnership status: (i) partner only; (ii) partner and others (e.g., adult children); (iii) no partner, with others; and (iv) living alone.

### Descriptive statistics

Table 2 shows the descriptive statistics. The first two columns present the means and percentages for individuals without (column 1) and with care needs (column 2), and columns (3) to (6) provide information for the different subsamples. Among those with care needs, 53.9% require fewer than 65 h of care per month^8^ and are therefore classified as non-eligible, whereas the remaining 46.1% meet the threshold and are classified as eligible. The “eligible” group is further stratified by severity of disability into two categories: individuals reporting fewer than two ADLs (28.8%) and those reporting two or more ADLs (71.2%).

**Table 2:** Sample descriptive statistics

Variables	With no care needs (N = 3,888)	With care needs (N = 3,306)	Non-eligible (N = 1,800)	Eligible (N = 1,506)	Eligible 0–1 ADLs (N = 434)	Eligible 2 + ADLs (N = 1,072)
	(1)	(2)	(3)	(4)	(5)	(6)
Female	46.05	69.08***	70.04	67.98	58.82	71.85***
*Age groups*						
65–69	36.49	13.99***	20.21	6.80***	7.83	6.37
70–74	29.39	18.05	23.49	11.76	10.32	12.37
75–79	19.84	18.78	21.37	15.80	16.54	15.48
80–84	8.43	20.33	19.33	21.49	24.73	20.12
85+	5.85	28.84	15.59	44.15	40.59	45.65
*Education*						
Low	67.78	84.75***	81.29	88.76***	89.90	88.27
Medium	14.78	7.18	9.07	5.00	5.24	4.89
High	17.44	8.07	9.64	6.25	4.86	6.83
*Living arrangements*						
With partner only	49.17	36.31***	40.55	31.41***	33.57	30.50
With partner and other	23.03	17.88	20.32	15.05	18.47	13.61
No partner but other	11.43	24.72	17.04	33.61	29.51	35.34
Living alone	16.37	21.09	22.10	19.93	18.44	20.55
Income	18,613 (7,148)	15,846 (7,148)***	16,390 (7,278)	15,218 (6,943)***	15,265 (6,896)	15,198 (6,966)
*Area of residence*						
Urban	81.21	76.29***	78.31	73.96**	78.83	72.44*
Rural	18.79	23.71	21.69	26.04	22.43	27.56
*Self-reported health*						
Less than good health	30.34	70.19***	60.08	87.65***	82.12	90.49***
Long term health problem	78.66	96.09***	93.95	98.56***	98.29	98.68
Number of chronic	1.83 (1.64)	3.37 (2.13)***	2.94 (1.95)	3.86 (2.22)***	3.64 (2.28)	3.95 (2.19)***
Number of ADLs	0 (0)	1.36 (1.84)***	0.09 (0.29)	2.83 (1.78)***	0.52 (0.50)	3.81 (1.10)***
Number of IADLs	0 (0)	3.09 (2.33)***	1.58 (0.98)	5.38 (1.76)***	5.09 (1.40)	5.47 (1.88)***

Values for categorical variables are in percent. For continuous variables, the mean values are shown, followed by standard errors in parenthesis. Sampling weights and design factors were accounted for when estimating prevalence/means. Individuals with under 65 h of LTC needs per month are classified as non-eligible, and those exceeding 65 h are classified as eligible. Columns (3) and (4) provide a subdivision of the group of individuals with care needs reported in column (2). Under living arrangement other indicates another household member different from the spouse

The Pearson´s chi-square test and the Student’s t-test are used to compare sample characteristic between groups (1 vs. 2, 3 vs.4 and 5 vs.6) for categorical and continuous variables, respectively

***p < 0.01; **p < 0.05; *p < 0.1

Individuals with care needs are, on average, older than those without care needs, and over two-thirds are women. They also have lower levels of education and income. Their health status is also poorer: 70.2% report less than good health (versus one in three among those without care needs), almost all have a long-term health condition (versus 78.7% among those without care needs), and report more than three chronic illnesses on average (compared with fewer than two in the no care needs group). Across eligibility groups, a similar pattern emerges. Care needs increase with age, especially among the oldest old (44.2% in the eligible group versus 15.6% among the non-eligible). Individuals with greater care needs also have significantly lower education and income levels, as well as poorer health. Among those eligible, differences across ADL-severity subgroups are smaller but still notable. Nearly eight in ten individuals with 2 + ADLs are women, compared with fewer than six in ten in the 0–1 ADL group. Their health status is also worse: 90.5% report less than good health (versus 82.1% in the lower-severity group), and they have more chronic conditions and a higher number of ADLs and IADLs.

Taken together, these descriptive findings reveal a clear gradient of vulnerability: greater care needs are associated with older age, lower socio-economic status, and worse health outcomes.

### Distribution of unmet LTC needs

Table 3 provides the distribution of unmet LTC needs among individuals identified as having care needs. Overall, 58.2% of these individuals have unmet needs, with 7.4% experiencing a full care gap. Unmet needs are twice as prevalent among those requiring more than 65 h of care per month (80%) compared with those with lower care needs (39%), largely driven by a greater partial gap. Non-eligible individuals exhibit the highest rate of fully unmet needs (9.3%), underscoring potential shortcomings in access to care among these individuals. Across ADL-severity groups, unmet needs, both partial and full, increase substantially with the level of limitations, with the most severely limited group facing roughly twice the full gap observed in those with fewer ADL limitations (6.1% vs. 3.0%).

**Table 3:** Distribution of met and unmet care needs by eligibility and disability severity

	With care needs (N = 3,306)	Non-eligible (N = 1,800)	Eligible (N = 1,506)	Eligible 0–1 ADLs (N = 434)	Eligible 2 + ADLs (N = 1,072)
No unmet needs (no gap)	41.84	60.78	19.96	29.91	15.77
Partially unmet needs (partial gap)	50.77	29.92	74.86	67.07	78.14
Fully unmet needs (full gap)	7.39	9.30	5.18	3.02	6.09

Individuals with under 65 h of LTC needs per month are classified as non-eligible, and those exceeding 65 h are classified as eligible

## Results

### Socio-economic factors contributing to unmet LTC needs

Before examining socio-economic inequality in unmet LTC needs in Spain, we first assess the distribution of unmet LTC needs in hours across sociodemographic groups and income quintiles (Table 4). Individuals with care needs have an average of 39.4 h of unmet LTC needs,^9^ ranging from 6.7 h in the non-eligible group to 94.2 h among those with two or more ADLs. Women consistently report higher unmet care hours than men, particularly in the eligible group (84.7 vs. 61.5) and among those with two or more ADLs (99.7 vs. 80). The care gap increases with age, peaking among individuals aged 85 + . Socio-economic gradients are evident: lower education and income are associated with significantly higher unmet needs. For example, eligible individuals in the bottom quintile have 89.2 unmet care hours, compared with 68.1 in the top quintile. Living arrangements show a strong protective effect, with individuals living alone experiencing the highest care gaps and those living with a partner the lowest (e.g., 121 vs. 64 h of unmet needs among those with two or more ADL limitations). A similar table depicting the distribution of LTC needs hours can be found in Table A1 in the Online Appendix.

**Table 4:** Average monthly hours of unmet LTC needs by socio-demographic characteristics, eligibility and disability severity

Variables	With care needs (N = 3,306)	Non-eligible (N = 1,800)	Eligible (N = 1,506)	Eligible 0–1 ADLs (N = 434)	Eligible 2 + ADLs (N = 1,072)
*Sex*					
Female	42.3 (1.5)***	6.6 (0.4)	84.7 (2.3)***	41.1 (2.3)***	99.7 (2.7)***
Male	33.1 (1.8)	6.8 (0.6)	61.5 (3.1)	31.5 (2.3)	80.0 (4.2)
*Age groups*					
65–69	18.3 (2.2)***	4.6 (0.7)***	65.4 (6.9)***	32.5 (5.5)	82.5 (8.8)***
70–74	27.3 (2.5)	5.9 (0.6)	76.4 (5.6)	35.1 (6.2)	91.0 (6.6)
75–79	30.6 (2.1)	6.9 (0.6)	67.5 (3.8)	36.0 (3.7)	81.7 (4.8)
80–84	38.7 (2.5)	8.1 (0.8)	70.4 (4.1)	33.8 (3.0)	89.4 (5.4)
85 +	63.6 (2.6)	8.2 (0.8)	86.1 (2.9)	41.1 (2.9)	103.1 (3.4)
*Education*					
Low	42.0 (1.3)***	7.0 (0.3)**	79.1 (2.0)***	37.6 (1.8)	97.0 (2.4)***
Medium	21.3 (2.7)	5.4 (0.8)	54.5 (6.5)	29.5 (6.5)	65.8 (8.2)
High	28.2 (3.9)	5.2 (1.0)	69.1 (8.1)	38.0 (7.8)	78.4 (9.8)
*Living arrangement*					
With partner only	24.2 (1.2)***	5.7 (0.4)***	51.9 (2.3)***	26.7 (2.2)***	63.5 (2.9)***
With partner and other	24.8 (2.3)	5.9 (0.8)	54.2 (4.2)	34.1 (3.7)	65.7 (5.6)
No partner but other	64.6 (3.3)	7.5 (0.9)	98.1 (3.7)	47.1 (3.7)	116.1 (4.1)
Living alone	48.4 (2.1)	8.4 (0.5)	99.1 (3.2)	43.4 (3.3)	121.1(3.7)
*Household income*					
1st quintile (lowest)	47.6 (3.7)***	7.1 (0.9)*	89.2 (5.1)***	39.8 (4.9)	109.1 (5.5)***
2nd quintile	45.4 (2.2)	7.5 (0.6)	79.1 (3.2)	35.0 (3.0)	96.5 (3.9)
3rd quintile	36.5 (2.3)	7.0 (0.6)	74.0 (4.0)	38.9 (3.8)	90.7 (5.1)
4th quintile	35.2 (2.8)	5.9 (0.7)	75.2 (4.7)	37.6 (3.6)	91.5 (5.8)
5th quintile (highest)	31.3 (2.6)	5.5 (0.7)	68.1 (4.8)	35.6 (4.2)	81.9 (6.1)
*Area of residence*					
Urban	39.04	6.94	78.74	37.58	97.34***
Rural	40.68	9.59	73.15	35.72	86.01
Total	39.4 (1.2)	6.7 (0.3)	77.3 (1.9)	37.2 (1.7)	94. 2 (2.3)

Standard errors in parenthesis; weighted results; one way ANOVA used for significance of contributing factors to the care gap

***p < 0.01; **p < 0.05; *p < 0.1

Individuals with under 65 h of LTC needs per month are classified as non-eligible, and those exceeding 65 h are classified as eligible

### Inequalities in unmet LTC needs and decomposition

Overall, our results indicate that unmet needs are disproportionately concentrated among the worse-off older people, as reflected by the negative CCI values in Table 5. Inequality is most pronounced among poorer individuals with severe limitations (CCI = − 0.062), suggesting that socio-economic disadvantage and high care needs compound to exacerbate unmet needs. By contrast, little to no inequality is observed among non-eligible individuals and among eligible individuals with only mild limitations (with CCI close to zero), indicating a more even distribution of unmet needs within these groups.

**Table 5:** Inequalities in unmet LTC needs across different subsamples of individuals aged 65 + with care needs in Spain (*Erreygers-*c*orrected Concentration Index*)

	With care needs (N = 3,306)	Non-eligible (N = 1,800)	Eligible (N = 1,506)	Eligible 0–1 ADLs (N = 434)	Eligible 2 + ADLs (N = 1,072)
CCI	− 0.050*** (− 0.070, − 0.030)	− 0.006*** (− 0.012, − 0.001)	− 0.046*** (− 0.077, − 0.010)	− 0.006 (− 0.036, 0.021)	− 0.062*** (− 0.098, − 0.025)

Bias-corrected bootstrapped 95% confidence intervals in parenthesis

***p < 0.01; **p < 0.05; *p < 0.1

Individuals with under 65 h of LTC needs per month are classified as non-eligible, and those exceeding 65 h are classified as eligible

**Fig. 2:**
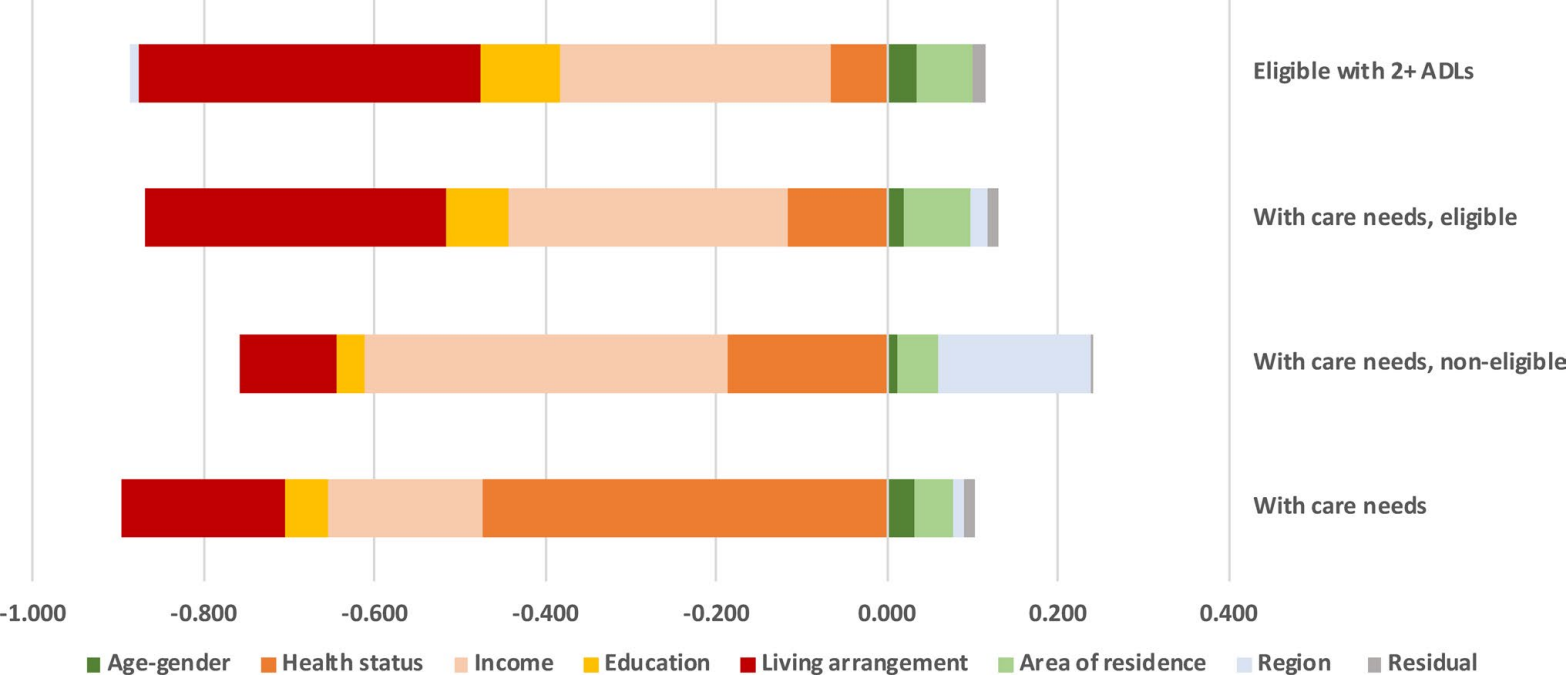
Decomposition of the CCI across different subsamples of individuals aged 65 + with care needs in Spain. Notes: Health status includes the number of chronic conditions and hours of care needs. Weighted results. Individuals with under 65 h of LTC needs per month are classified as non-eligible, and those exceeding 65 h are classified as eligible. Variables depicted in the left-hand side of 0 contribute to inequality in unmet LTC needs disfavouring the poor; variables depicted on the right-hand side of 0 contribute to pro-rich inequality. Reading: income, for example, contributes to inequality by -0.020 (out of a total inequality index of -0.046) among older eligible individuals with care needs.

To better understand the determinants underlying the observed inequalities, we perform a decomposition analysis of the CCI. This method allows to quantify the contribution of each explanatory variable to the overall income-related inequality in unmet needs. Figure 2 presents the results of the decomposition of CCI, showing the absolute contributions of the different groups of determinants. A detailed breakdown of each factor’s contribution, reported in terms of its corresponding semi-elasticity and concentration index, separately for each subsample, is provided in Tables A2–A5 (Online appendix).

Results indicate that the main contributors to income-related inequality in unmet needs are health status, income and living arrangements, although their relative influence varies across subsamples. Education and area of residence also contribute to the unequal concentration of unmet care needs, albeit to a lesser extent.

Household structure and socio-economic characteristics are the primary drivers of inequalities in unmet needs disfavouring the poor. For example, we observe that living alone is more common among the worse off (CI = − 0.166) and is associated with increases in unmet needs (semi-elasticity = 4.49), resulting in a substantial negative contribution to overall inequality (Table A2). This effect is particularly pronounced among those with greater limitations (with CI = − 0.211 and semi-elasticity = 12.08) (Table A5). The contribution of income in the overall sample is also negative, due to unmet needs being more prevalent among the poor (semi-elasticity = − 3.02). Higher educational levels are concentrated among wealthier individuals (CI = 0.477) and are inversely related to unmet LTC needs (semi-elasticity = − 0.31), thereby also contributing negatively to total inequality. These patterns are consistent across all subsamples. Health status also contributes to the unequal distribution of unmet needs. Overall, poor health is concentrated among lower-income individuals (e.g., CI = − 0.061 for number of ADLs) and is associated with greater unmet needs (e.g., semi-elasticity = 22.62 for number of ADLs) (Table A2). The area of residence contributes positively to inequality. Lower-income individuals tend to reside in rural areas (CI = − 0.140), yet rural living is linked to lower unmet LTC needs (semi-elasticity = − 1.22).

### Inequity in unmet LTC needs

Table 6 presents the inequity indices across subsamples. The overall negative value of − 0.022 indicates that, after adjusting for differences in need, the distribution of unmet LTC needs remains concentrated among the poor. The CCI_non-need_ is highest among those with severe limitations ( − 0.066), while no inequity emerges for either the non-eligible group or eligible individuals with mild limitations.

**Table 6:** Inequity in unmet LTC needs across different subsamples of individuals aged 65 + with care needs in Spain

	With care needs (N = 3,306)	Non-eligible (N = 1,800)	Eligible (N = 1,506)	Eligible 0–1 ADLs (N = 434)	Eligible 2 + ADLs (N = 1,072)
CCI_non-need_	− 0.022*** (− 0.035, − 0.009)	− 0.004* (− 0.009, 0.001)	− 0.040*** (− 0.066, − 0.017)	− 0.004 (− 0.020, 0.027)	− 0.066*** (− 0.103, − 0.029)

Bias-corrected bootstrapped 95% confidence intervals in parenthesis

***p < 0.01; **p < 0.05; *p < 0.1

Individuals with under 65 h of LTC needs per month are classified as non-eligible, and those exceeding 65 h are classified as eligible

## Discussion

This study extends existing research on unmet LTC needs by presenting new evidence on their prevalence, socio-economic inequality and underlying drivers. We achieve this through a novel operationalisation of the unmet needs that combines information on ADLs, IADLs, the absence of care, and the perceived adequacy of received care into a single indicator, which we then map into hours of unmet LTC needs using a replicable approach. This strengthens the evidence base for policymaking.

We find substantial unmet LTC needs among older adults, with 50.8% of individuals with care needs lacking adequate support, and 7.4% receiving no help at all. Our prevalence estimates are similar or even higher than those reported in previous research on Spain (e.g., Albuquerque 2022; Calderón-Jaramillo and Zueras 2023), most likely reflecting variations in the definition of the ‘at-risk’ population and in the measurement of unmet needs. Women consistently report higher unmet LTC needs than men, pointing to a disproportionate burden on women. This result is in line with previous findings for Spain (Hernández-Quevedo and Jiménez Rubio 2011; Albuquerque 2022). It also aligns with evidence from the UK, where women are found to report more subjective unmet needs than men, possibly because they have higher expectations of care and are more likely to perceive it as insufficient (Zhang et al. 2025). The care gap widens with age, reflecting the cumulative vulnerabilities of the oldest old (Kröger et al. 2019; Albuquerque 2022). Living arrangements show a strong protective effect against unmet needs, with individuals living alone experiencing the highest care gaps, and those living with a partner the lowest (Dunatchik et al. 2019; Szenkurök et al. 2024).

We show clear evidence of inequality and inequity in LTC access in terms of unmet needs, confirming previous findings from Spain (Hernández-Quevedo and Jiménez Rubio 2011; García-Gómez et al. 2015). In particular, unmet needs are disproportionately concentrated among lower-income adults aged 65 and over, with the greatest disparities observed among individuals with severe limitations (2 + ADLs). Inequality in unmet LTC needs disfavouring the poor may arise because older adults in lower socio-economic groups face multiple access barriers. These include a lower ability to afford care, especially private services (Carrieri et al. 2017), or limited availability of public provision due to waiting lists or insufficient supply (Hernández-Quevedo and Jiménez Rubio 2011). They also tend to rely more heavily on informal support (García-Gómez et al 2015, Serrano-Alarcón and Perelman 2017), which is often weak or insufficient (Beach and Schulz 2017), and may struggle to navigate a complex care system due to lower educational attainment (Albuquerque 2022; Okamoto et al. 2025).

Our decomposition analysis indicates that income, living arrangements, and health status are the key contributors to socio-economic inequality in unmet LTC needs among older adults in Spain. To our knowledge, only one study has applied a decomposition approach to inequality in unmet LTC needs for the UK (Hu et al. 2022), and it reports similar results. Decomposition analyses in the literature on inequality in LTC use also find that income, education and household composition are the main determinants of inequality (Ilinca et al. 2017; Carrieri et al. 2017; Tenand et al. 2020).

Our analysis has several limitations. First, to quantify LTC needs in Spain, we apply the Austrian LTC assessment scheme, which maps limitations with personal care, household tasks and mental health into hours of care needed. Although not ideal, this approach relies on a reasonable assumption that identical limitations imply similar care needs across countries. We do not impose any assumptions about the source of care provision and financing. Furthermore, we acknowledge that classifying individuals into eligible and non-eligible groups based on the 65-h threshold may not perfectly reflect the actual distribution of care needs or eligibility rules in Spain; however, it serves solely as a means to label and distinguish between those with mild and severe limitations. Second, older individuals residing in nursing homes are excluded from the survey. Since those with the greatest LTC needs, and plausibly higher levels of unmet needs, are more likely to be institutionalised, this omission may introduce selection bias in our estimates. Third, we use annual equivalised household income as ranking variable rather than wealth. Although older individuals experience little variation in annual income, mainly based on old-age pensions, their wealth (real estate) varies considerably. Several studies compare inequity in LTC using both wealth and income (Rodrigues et al. 2018; Lera et al. 2021) and generally find that, in Spain, inequity in the use of both formal and informal care is similar regardless of the ranking variable. An exception is Rodrigues et al. (2018), who report that using wealth accentuates the concentration of informal care among poorer individuals. Additionally, household income may reflect care-driven living arrangements or include cash benefits for informal carers, however, this cannot be corrected with EHIS data. Fourth, our analysis is based on self-reported data, which are subject to well-known limitations. Fifth, the study is cross-sectional, providing only a snapshot of LTC inequalities in Spain. Future research using longitudinal data could help assess the impact of policy changes in the LTC system on the evolution of these inequalities.

Despite these limitations, our study offers a rigorous quantification of unmet LTC needs and uncovers important socio-economic inequities in Spain, providing evidence that can inform policy responses to rising care demands and limited supply in ageing societies.

## Conclusions

Our paper deepens the understanding of unmet LTC needs among older people in Spain and contributes to the growing but still limited research on inequality and inequity in this area. Significant socio-economic inequalities and inequities in unmet LTC needs are observed among individuals with care needs, driven by the disproportionate concentration of unmet needs among the most disadvantaged older adults with severe limitations. The strong contributions of income, education, and living arrangements to inequality disfavouring the poor suggest that financial constraints and limited informal support are central drivers of unmet needs. Expanding financial protection for low-income households, strengthening community-based services for people living alone, and targeted outreach to vulnerable groups could help mitigate these disparities. Moreover, the sizable contribution of health status points to the need to better integrate LTC with preventive and primary care, in order to address complex health needs and prevent progression to higher dependency.

## Supplementary Information

Below is the link to the electronic supplementary material.

Supplementary file1 (DOCX 51 KB)

Supplementary file2 (DOCX 90 KB)

## Data Availability

This study uses secondary data from the European Health Interview Survey (EHIS).

## Footnotes

1 Similar, but broader concepts have recently been introduced in research on unmet needs, namely the concept of care poverty—defined as the interaction between older individuals’ characteristics and wider societal factors that shape the availability and distribution of care services—which distinguishes between absolute care poverty, the complete lack of necessary help, and relative care poverty, situations of insufficient support (Kröger et al. 2019; Kröger 2022; Kröger et al. 2025).

2 The official recognition of dependency status does not necessarily translate into receipt of services or benefits. In practice, there are often considerable delays at multiple stages of the process, including both the initial assessment and recognition of dependency, and the subsequent allocation and provision of benefits. These delays have resulted in substantial waiting lists, which undermine the system’s ability to guarantee timely support and may disproportionately affect individuals with urgent care needs.

3 Each limitation is assigned a fixed number of hours, which are then summed to determine eligibility, starting at 65 h per month, with higher allowances for greater needs.

4 To identify individuals who are severely limited due to mental health problems, we use responses to two survey questions: (i) ‘*Are you limited because of a health problem in the activities people usually do for at least the past six months?*’. For respondents indicating that they are ‘severely limited,’ we then consider (ii) *‘What type of problem is causing your difficulty in performing activities people usually do?’* and select those who report a mental health problem.

5 Unmet care needs are set as: 25% of LTC needs (in hours) for individuals living with a spouse and receiving formal care; 50% for those living with a spouse and not receiving formal care or living with another adult and receiving formal care; 75% for those living alone and receiving formal care or living with another adult and not receiving formal care; and 85% for those living alone and not receiving formal care.

6 Details are available from the authors upon request.

7 Further details on the imputation of the income variable can be found in the Supplementary material.

8 The Austrian LTC system establishes an eligibility threshold of 65 h of care per month for LTC support. For consistency with the LTC need-mapping approach, we applied this same cutoff to classify individuals with care needs as either eligible and non-eligible.

9 The distribution of unmet LTC needs ranges from 0 to 268 h, corresponding to the a_n_ and b_n_ bounds used in the Erreygers’ correction applied in Eqs. (2) and (4) in the Methodology. The variable is truncated at 0 and present substantial positive asymmetry (skewness = 1.42).
