# Supplementary material for: Socio-economic inequalities in unmet long-term care needs in Spain
Source: Eur J Ageing. 2026 Feb 4;23(1):11. doi: 10.1007/s10433-026-00910-3 (PMC12965937; doi:10.1007/s10433-026-00910-3)
Supplement: Supplementary file 1 — Supplementary file1 (DOCX 51 KB) [file 10433_2026_910_MOESM1_ESM.docx]

**Online Appendix**

Article title: **Socioeconomic Inequality in Unmet Long-Term Care Needs**

Authors: Raquel Andres and Alexandrina Stoyanova

Journal: European Journal of Ageing

Table A1 Average monthly hours of unmet LTC needs by socio-demographic characteristics, eligibility and disability severity

|  | With care needs  (N=3,306) | W/ care needs, non-eligible  (N=1,800) | W/ care needs, eligible  (N=1,506) | Eligible  0-1 ADLs  (N=434) | Eligible  2+ ADLs  (N=1,072) |
| --- | --- | --- | --- | --- | --- |
| *Sex* |  |  |  |  |  |
| Female | 82.7 (3.0) | 21.1 (0.5)^***^ | 162.1 (2.6)^***^ | 89.1 (1.6)^*^ | 187.3(2.4) |
| Male | 85.4 (2.3) | 24.4 (0.8) | 145.7 (3.7) | 85.3 (2.1) | 183.0 (3.9) |
| *Age groups* |  |  |  |  |  |
| 65-69 | 46.1 (3.7)^***^ | 18.8 (0.9)^***^ | 139.7 (8.6)^***^ | 81.1 (4.2)^***^ | 170.2 (8.5)^***^ |
| 70-74 | 59.2 (3.9) | 19.1 (0.8) | 151.7 (6.7) | 81.7 (3.0) | 176.4 (6.9) |
| 75-79 | 70.0 (3.5) | 22.8 (0.9) | 143.8 (4.6) | 89.2 (3.2) | 168.4 (4.9) |
| 80-84 | 85.5 (3.7) | 24.3 (0.9) | 149.2 (4.5) | 83.7 (2.4) | 183.1 (4.6) |
| 85+ | 128.0 (3.5) | 27.1 (1.2) | 169.2 (3.1) | 91.9 (2.1) | 198.2 (2.7) |
| *Education* |  |  |  |  |  |
| Low | 87.9 (2.0)^***^ | 22.6 (0.5)^***^ | 157.0 (2.2)^**^ | 87.3 (1.3)^**^ | 187.0 (2.1)^**^ |
| Medium | 58.2 (5.4) | 19.2 (1.2) | 140.1 (8.8) | 81.8 (4.2) | 166.4 (8.9) |
| High | 73.4 (6.7) | 20.6 (1.6) | 167.5 (9.2) | 98.3 (7.7) | 188.3 (10.0) |
| *Living arrangement* |  |  |  |  |  |
| With partner only | 73.8 (2.6)^***^ | 22.1 (0.6) | 151.0 (3.5)^***^ | 87.1 (2.1) | 180.6 (3.7)^***^ |
| With partner and other | 72.8 (5.2) | 21.9 (1.2) | 152.3 (7.3) | 87.1 (4.0) | 189.7 (7.2) |
| No partner but other | 114.1 (4.3) | 22.6 (1.0) | 167.7 (3.9) | 89.9 (2.4) | 195.1 (3.4) |
| Living alone | 78.5 (2.4) | 21.8 (0.6) | 151.1 (2.9) | 84.8 (1.7) | 176.2 (3.1) |
| *Household income* |  |  |  |  |  |
| 1^st^ quintile | 93.1 (5.1)^***^ | 22.1 (1.1)^*^ | 166.0 (5.3)^*^ | 90.6 (2.6) | 196.4 (4.3)^***^ |
| 2^nd^ quintile | 92.8 (3.4) | 23.2 (0.8) | 154.8 (3.6) | 85.9 (2.5) | 182.0 (3.5) |
| 3^rd^ quintile | 79.5 (3.5) | 22.8 (0.9) | 151.5 (4.3) | 90.7 (2.6) | 180.3 (4.6) |
| 4^th^ quintile | 78.0 (4.3) | 21.5 (0.9) | 155.2 (5.4) | 84.1 (2.8) | 186.0 (5.1) |
| 5^th^ quintile | 78.8(4.8) | 20.3 (1.1) | 161.9 (6.5) | 87.0 (3.7) | 186.0 (5.1) |
| *Area of residence* |  |  |  |  |  |
| Urban | 82.9 (2.1)^**^ | 22.2 (0.5) | 157.1 (2.5) | 88.0 (1.5) | 188.3 (2.4)^**^ |
| Rural | 90.2 (3.8) | 21.7 (0.9) | 156.1 (3.9) | 85.7 (2.6) | 180.3 (3.8) |
| Total | 84.6 (1.8) | 22.1 (0.4) | 156.8 (2.1) | 87.5 (1.3) | 186.1 (2.0) |

Notes: Standard errors in parenthesis; weighted results; one way ANOVA used for significance of contributing factors to the care gap; ^***^p<0.01; ^**^p<0.05; ^*^ p<0.1.

Individuals with under 65 hours of LTC needs per month are classified as non-eligible, and those exceeding 65 hours are classified as eligible.

Table A2 Decomposition of the CCI for unmet LTC needs among individuals with care needs

|  | Semi-elasticity | CI | Absolute  Contribution | Relative  Contribution (%) |
| --- | --- | --- | --- | --- |
| **Need variables** |  |  |  |  |
| Female | 0.877 | -0.012^*^ | -0.0002 | 0.30 |
| 70-74 years old | 0.297 | 0.087^***^ | 0.0004 | -0.77 |
| 75-79 years old | -0.601^*^ | -0.040^*^ | 0.0004 | -0.71 |
| 80-84 years old | -0.917^**^ | -0.043^**^ | 0.0006 | -1.17 |
| 85+ years old | -1.555^**^ | -0.036^**^ | 0.0008 | -1.66 |
| Num. of chronic conditions | -2.315^*^ | -0.011^*^ | 0.0004 | -0.76 |
| Number of ADLs | 22.615^***^ | -0.061^***^ | -0.0207 | 41.33 |
| Number of IADLs | 21.131^***^ | -0.030^***^ | -0.0094 | 18.83 |
| Long-term health problem | 4.079 | -0.003 | -0.0002 | 0.35 |
| **Total need** |  |  | **-0.0280** | **55.74** |
|  |  |  |  |  |
| **Non-need** |  |  |  |  |
| Household income | -3.022^*^ | 0.252^***^ | -0.0114 | 22.69 |
| Medium education | -0.278^*^ | 0.260^***^ | -0.0011 | 2.15 |
| High education | -0.305 | 0.477^***^ | -0.0022 | 4.33 |
| Partner and other | -0.017 | 0.157^***^ | 0.0000 | 0.08 |
| No partner but other | 4.852^***^ | -0.012 | -0.0009 | 1.80 |
| Living alone | 4.487^***^ | -0.166^***^ | -0.0111 | 22.18 |
| Towns and suburban | -0.511 | -0.045^***^ | 0.0003 | -0.68 |
| Rural | -1.217^***^ | -0.140^***^ | 0.0025 | -5.06 |
| Aragon | 0.069 | -0.160^***^ | -0.0002 | 0.33 |
| Asturias | 0.015 | 0.093 | 0.0000 | -0.04 |
| Balearic Islands | -0.126 | -0.030 | 0.0001 | -0.11 |
| Canary Islands | -0.358^*^ | -0.165^***^ | 0.0009 | -1.76 |
| Cantabria | -0.035 | 0.119 | -0.0001 | 0.12 |
| Castilla-Leon | -0.107 | -0.053 | 0.0001 | -0.17 |
| Castilla la Mancha | -0.278 | -0.079^**^ | 0.0003 | -0.66 |
| Catalonia | 0.364 | 0.144^***^ | 0.0008 | -1.56 |
| Valencia | -0.382 | -0.003 | 0.0000 | -0.04 |
| Extremadura | -0.015 | -0.132^**^ | 0.0000 | -0.06 |
| Galicia | 0.298 | 0.098^***^ | 0.0004 | -0.87 |
| Madrid | -0.818^**^ | 0.154^***^ | -0.0019 | 3.76 |
| Murcia | -0.203 | -0.015 | 0.0000 | -0.09 |
| Navarra | -0.047 | 0.030 | 0.0000 | 0.04 |
| Basque Country | 0.201 | 0.102^**^ | 0.0003 | -0.61 |
| Rioja | -0.036 | 0.074 | 0.0000 | 0.08 |
| Ceuta y Melilla | -0.022 | 0.157 | -0.0001 | 0.10 |
| **Total non-need** |  |  | **-0.0231** | **45.95** |
| *Residual* |  |  | *0.0008* | *-1.69* |

Notes: Weighted results; ^***^p<0.01; ^**^p<0.05; ^*^ p<0.1.

Individuals with under 65 hours of LTC needs per month are classified as non-eligible, and those exceeding 65 hours are classified as eligible.

Table A3 Decomposition of the CCI for unmet LTC needs among non-eligible individuals

|  | Semi-elasticity | CI | Absolute  Contribution | Relative  Contribution (%) |
| --- | --- | --- | --- | --- |
| **Need variables** |  |  |  |  |
| Female | -0.082 | -0.001 | 0.0000 | -0.03 |
| 70-74 years old | 0.154 | 0.094^***^ | 0.0002 | -3.60 |
| 75-79 years old | 0.015 | -0.074^***^ | 0.0000 | 0.27 |
| 80-84 years old | 0.104 | -0.052^*^ | -0.0001 | 1.34 |
| 85+ years old | -0.087 | -0.016 | 0.0000 | -0.35 |
| Num. of chronic conditions | 1.193^**^ | -0.023^**^ | -0.0004 | 6.85 |
| Number of ADLs | 0.957^***^ | -0.121^***^ | -0.0017 | 28.77 |
| Number of IADLs | 7.370^***^ | -0.001 | -0.0001 | 1.53 |
| Long-term health problem | -0.528 | -0.006 | 0.0001 | -0.84 |
| **Total need** |  |  | **-0.0020** | **33.94** |
|  |  |  |  |  |
| **Non-need** |  |  |  |  |
| Household income | -1.324^**^ | 0.249^***^ | -0.0049 | 81.98 |
| Medium education | -0.035 | 0.243^***^ | -0.0001 | 2.11 |
| High education | -0.037 | 0.477^***^ | -0.0002 | 4.06 |
| Partner and other | 0.016 | 0.148^***^ | 0.0000 | -0.57 |
| No partner but other | 0.280^*^ | -0.035 | -0.0001 | 2.47 |
| Living alone | 0.566 | -0.143^***^ | -0.0012 | 20.12 |
| Towns and suburban | -0.246 | -0.102^***^ | 0.0004 | -6.25 |
| Rural | -0.103 | -0.107^***^ | 0.0002 | -2.73 |
| Aragon | 0.103^**^ | -0.132^*^ | -0.0002 | 3.39 |
| Asturias | 0.087^*^ | 0.062 | 0.0001 | -1.33 |
| Balearic Islands | -0.037^*^ | -0.325^**^ | 0.0002 | -3.01 |
| Canary Islands | -0.125 | -0.119^***^ | 0.0002 | -3.70 |
| Cantabria | -0.014 | 0.012 | 0.0000 | -0.04 |
| Castilla-Leon | -0.045 | -0.146^***^ | 0.0001 | -1.64 |
| Castilla la Mancha | 0.006 | -0.036 | 0.0000 | 0.05 |
| Catalonia | 0.284 | 0.160^***^ | 0.0007 | -11.30 |
| Valencia | -0.015 | -0.050 | 0.0000 | -0.19 |
| Extremadura | 0.020 | -0.093 | 0.0000 | 0.47 |
| Galicia | 0.319^**^ | 0.106^**^ | 0.0005 | -8.42 |
| Madrid | 0.053 | 0.146^***^ | 0.0001 | -1.93 |
| Murcia | -0.004 | -0.043 | 0.0000 | -0.04 |
| Navarra | 0.008 | -0.189 | 0.0000 | 0.36 |
| Basque Country | 0.270^***^ | 0.087^*^ | 0.0004 | -5.86 |
| Rioja | 0.022^***^ | 0.159 | 0.0001 | -0.89 |
| Ceuta y Melilla | 0.006 | 0.299 | 0.0000 | -0.46 |
| **Total non-need** |  |  | **-0.0040** | **66.66** |
| *Residual* |  |  | *0.0000* | *-0.60* |

Notes: Weighted results; ^***^p<0.01; ^**^p<0.05; ^*^ p<0.1.

Individuals with under 65 hours of LTC needs per month are classified as non-eligible, and those exceeding 65 hours are classified as eligible.

Table A4 Decomposition of the CCI for unmet LTC needs among eligible individuals

|  | Semi-elasticity | CI | Absolute  Contribution | Relative  Contribution (%) |
| --- | --- | --- | --- | --- |
| **Need variables** |  |  |  |  |
| Female | 0.520 | -0.025^**^ | -0.0002 | 0.42 |
| 70-74 years old | 0.604 | 0.008 | 0.0001 | -0.17 |
| 75-79 years old | -0.972 | -0.009 | 0.0001 | -0.30 |
| 80-84 years old | -1.437 | -0.030 | 0.0006 | -1.41 |
| 85+ years old | -4.344^*^ | -0.007 | 0.0005 | -1.03 |
| Num. of chronic conditions | -5.100^**^ | 0.013 | -0.0010 | 2.15 |
| Number of ADLs | 46.257^***^ | -0.008 | -0.0058 | 12.68 |
| Number of IADLs | 35.367^***^ | -0.003 | -0.0015 | 3.35 |
| Long-term health problem | 20.338^*^ | 0.004^**^ | 0.0011 | -2.44 |
| **Total need** |  |  | **-0.0061** | **13.25** |
|  |  |  |  |  |
| **Non-need** |  |  |  |  |
| Household income | -5.360^*^ | 0.254^***^ | -0.0204 | 44.30 |
| Medium education | -0.344 | 0.255^***^ | -0.0013 | 2.85 |
| High education | -0.438 | 0.500^***^ | -0.0033 | 7.11 |
| Partner and other | 0.044 | 0.152^***^ | 0.0001 | -0.22 |
| No partner but other | 12.399^***^ | 0.029 | 0.0053 | -11.54 |
| Living alone | 9.183^***^ | -0.199^***^ | -0.0273 | 59.43 |
| Towns and suburban | -0.771 | 0.020 | -0.0002 | 0.51 |
| Rural | -2.135^**^ | -0.161^***^ | 0.0051 | -11.17 |
| Aragon | 0.045 | -0.198^**^ | -0.0001 | 0.29 |
| Asturias | 0.034 | 0.128 | 0.0001 | -0.14 |
| Balearic Islands | -0.275 | 0.143 | -0.0006 | 1.28 |
| Canary Islands | -0.650^*^ | -0.276^***^ | 0.0027 | -5.84 |
| Cantabria | -0.044 | 0.249 | -0.0002 | 0.36 |
| Castilla-Leon | -0.045 | 0.056 | 0.0000 | 0.08 |
| Castilla la Mancha | -0.527 | -0.169^**^ | 0.0013 | -2.89 |
| Catalonia | 0.586 | 0.106^**^ | 0.0009 | -2.02 |
| Valencia | -0.593 | 0.051 | -0.0005 | 0.98 |
| Extremadura | -0.051 | -0.154^*^ | 0.0001 | -0.25 |
| Galicia | 0.382 | 0.102^**^ | 0.0006 | -1.27 |
| Madrid | -1.232^**^ | 0.165^***^ | -0.0030 | 6.60 |
| Murcia | -0.337 | 0.018 | -0.0001 | 0.20 |
| Navarra | -0.021 | 0.239^*^ | -0.0001 | 0.16 |
| Basque Country | 0.234 | 0.083 | 0.0003 | -0.63 |
| Rioja | -0.079 | 0.005 | 0.0000 | 0.01 |
| Ceuta y Melilla | -0.062 | 0.105 | -0.0001 | 0.21 |
| **Total non-need** |  |  | **-0.0406** | **88.39** |
| *Residual* |  |  | *0.0008* | *-1.64* |

Notes: Weighted results; ^***^p<0.01; ^**^p<0.05; ^*^ p<0.1.

Individuals with under 65 hours of LTC needs per month are classified as non-eligible, and those exceeding 65 hours are classified as eligible.

Table A5 Decomposition of the CCI for unmet LTC needs among eligible individuals with 2+ ADLs

|  | Semi-elasticity | CI | Absolute  Contribution | Relative  Contribution (%) |
| --- | --- | --- | --- | --- |
| **Need variables** |  |  |  |  |
| Female | -0.576 | -0.017 | 0.0002 | -0.24 |
| 70-74 years old | 2.047^**^ | 0.084^*^ | 0.0026 | -4.15 |
| 75-79 years old | -0.201 | -0.038 | 0.0001 | -0.18 |
| 80-84 years old | -0.221 | -0.056 | 0.0002 | -0.30 |
| 85+ years old | -2.012 | 0.009 | -0.0003 | 0.44 |
| Num. of chronic conditions | -8.039^**^ | 0.010 | -0.0012 | 1.92 |
| Number of ADLs | 72.433^***^ | -0.004 | -0.0042 | 6.82 |
| Number of IADLs | 31.398^***^ | -0.001 | -0.0004 | 0.58 |
| Long-term health problem | 22.284 | 0.002 | 0.0005 | -0.87 |
| **Total need** |  | **-0.017** | **-0.0025** | **4.01** |
|  |  |  |  |  |
| **Non-need variables** |  |  |  |  |
| Household income | -6.678 | 0.255^***^ | -0.0254 | 41.33 |
| Medium education | -0.575 | 0.211^***^ | -0.0018 | 2.94 |
| High education | -0.721 | 0.513^***^ | -0.0055 | 8.97 |
| Partner and other | -0.446 | 0.139^***^ | -0.0009 | 1.50 |
| No partner but other | 16.542^***^ | 0.029 | 0.0071 | -11.46 |
| Living alone | 12.077^***^ | -0.211^***^ | -0.0381 | 61.84 |
| Towns and suburban | -0.220 | 0.061^**^ | -0.0002 | 0.33 |
| Rural | -2.224^*^ | -0.166^***^ | 0.0055 | -8.97 |
| Aragon | 0.045 | -0.215^*^ | -0.0001 | 0.23 |
| Asturias | 0.093 | 0.092 | 0.0001 | -0.21 |
| Balearic Islands | -0.365 | 0.365^***^ | -0.0020 | 3.24 |
| Canary Islands | -0.574 | -0.298^***^ | 0.0025 | -4.14 |
| Cantabria | -0.105 | 0.269^*^ | -0.0004 | 0.69 |
| Castilla-Leon | -0.151 | 0.035 | -0.0001 | 0.13 |
| Castilla la Mancha | -0.417 | -0.138^*^ | 0.0009 | -1.40 |
| Catalonia | 0.359 | 0.091^*^ | 0.0005 | -0.79 |
| Valencia | -0.785 | 0.057 | -0.0007 | 1.09 |
| Extremadura | -0.247 | -0.132 | 0.0005 | -0.79 |
| Galicia | 0.397 | 0.072 | 0.0004 | -0.70 |
| Madrid | -0.948 | 0.174^***^ | -0.0025 | 4.00 |
| Murcia | -0.597^*^ | 0.039 | -0.0003 | 0.56 |
| Navarra | -0.087 | 0.158 | -0.0002 | 0.33 |
| Basque Country | 0.313 | 0.177^**^ | 0.0008 | -1.35 |
| Rioja | -0.111 | 0.075 | -0.0001 | 0.20 |
| Ceuta y Melilla | -0.102 | 0.093 | -0.0001 | 0.23 |
| **Total non-need** |  |  | **-0.0602** | **97.80** |
| *Residual* |  |  | *0.0011* | *-1.81* |

Notes: Weighted results; ^***^p<0.01; ^**^p<0.05; ^*^ p<0.1.

Individuals with under 65 hours of LTC needs per month are classified as non-eligible, and those exceeding 65 hours are classified as eligible.
