# Supplementary material for: Socio-economic inequalities in unmet long-term care needs in Spain
Source: Eur J Ageing. 2026 Feb 4;23(1):11. doi: 10.1007/s10433-026-00910-3 (PMC12965937; doi:10.1007/s10433-026-00910-3)
Supplement: Supplementary file 2 — Supplementary file2 (DOCX 90 KB) [file 10433_2026_910_MOESM2_ESM.docx]

**Supplementary Material. Imputation of Continuous Income Variable**

This supplementary material describes the imputation procedure for the income variable, which is originally reported in quintiles in the 2019 European Health Interview Survey (EHIS) for Spain. Figure S1 shows the distribution of the income variable as it appears in the data for the sample of individuals with care needs.

Fig. S1 Distribution of income quintiles in the subsample of individuals with care needs in Spain


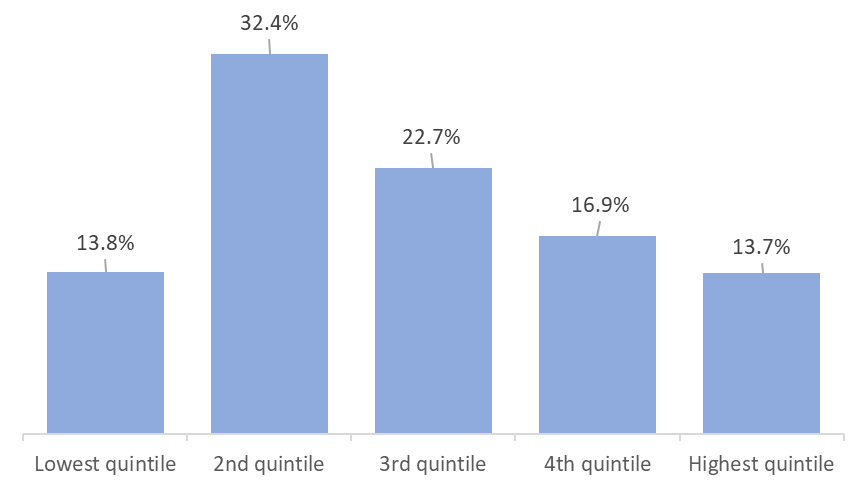


Note: Data comes from the Spanish sample of the EHIS 2019. The sample of individuals with care needs includes those who report difficulties with at least one ADL or IADL.

To obtain continuous income values from the categorical income quintiles in EHIS 2019, we estimate an interval regression model using lower- and upper-income cut-offs derived from the equivalised disposable income from the European Union Statistics on Income and Living Conditions (EU-SILC) 2019 cross-sectional microdata (Eurostat 2019) for Spain. For each EHIS income quintile, we assigned the corresponding lower and upper bounds of the EU-SILC equivalised income distribution and treated these as censoring points in the model.

The interval regression assumes an underlying latent continuous income variable that follows a normal distribution conditional on covariates (age, sex, education, degree of urbanisation, labour status, household type, and region of residence). After estimating the model, rather than assigning expected values within each interval—which would produce clustered and discretised predictions—we generate a random draw from the model-implied truncated normal distribution for each individual. For each observation, we use the estimated linear predictor and residual standard deviation to calculate the truncated bounds of the latent income distribution, compute the cumulative distribution at those bounds, draw a uniform random number, and apply the inverse normal transformation to obtain a simulated latent income value within the appropriate interval.

This approach produces a smooth and realistic continuous income distribution while remaining consistent with the observed quintile intervals in EHIS and the EU-SILC based interval-regression model.

Figure S2 shows the original income distribution observed in EU-SILC and compares it to the imputed income to the EHIS sample. The imputed income distribution (right panel) displays a somewhat thinner right tail compared with the original EU-SILC distribution (left panel). This pattern is expected given the structure of the EHIS income variable, where the highest income category is open-ended and therefore only bounded below. Interval regression estimates the latent continuous income under a normality assumption and uses information from all income intervals to infer overall dispersion. Because the upper interval provides no information on the extent of variation among high-income households, the model cannot reproduce the full degree of right-tail inequality observed in EU-SILC. As a result, extreme high-income values tend to be compressed toward the lower part of the top interval. This does not affect the central part of the distribution, which is well recovered, but it naturally leads to a less pronounced upper tail in the imputed data.

Fig. S2 Equivalent income distributions in EU-SILC (original) and EHIS (imputed)

|  |  |
| --- | --- |

Notes: Data comes from the Spanish sample of the EU-SILC and the EHIS 2019.
